# Supplementary material for: barCoder: a tool to generate unique, orthogonal genetic tags for qPCR detection
Source: BMC Bioinformatics. 2021 Mar 1;22:98. doi: 10.1186/s12859-021-04019-5 (PMC7919090; doi:10.1186/s12859-021-04019-5)
Supplement: Supplementary file 1 — Additional file 1: Tables S1. Sequences for the 21 barcodes designed with the barCoder algorithm. Table S2. Primer and probe sequences for each barcode module. Table S3. Details the constraint properties of the primers and probes that were experimentally validated in this study. Table S4. Details the check results for properties of the primers and probes that were experimentally validated in this study. Table S5. Details the BLAST check results for the primers and probes that were experimentally validated in this study. Table S6. Details the spacer and barcode module checks for the barcodes experimentally validated in this study. Table S7. Sources of DNA used in the cross-reactivity panel shown in Table 4. Table S8. Sequences of the primers used to construct the barcoded strain of Y. pestis CO92 pgm−. Figure S1. Additional qPCR standard curves. [file 12859_2021_4019_MOESM1_ESM.docx]

**Supplementary Tables and Figure**

**barCoder: a tool to generate unique, orthogonal genetic tags for qPCR detection**

Casey B. Bernhards^1,2*^, Matthew W. Lux^1*^, Sarah E. Katoski^1^, Tyler D. P. Goralski^1^, Alvin T. Liem^1,3^, Henry S. Gibbons^1†^

^*^These authors contributed equally to this work

^†^Corresponding author

^1^U.S. Army Combat Capabilities Development Command Chemical Biological Center, Aberdeen Proving Ground, MD 21010, USA

^2^Excet, Inc., Springfield, VA 22150, USA

^3^DCS Corporation, Abingdon, MD 21009, USA

Table S1. Barcode sequences.

| **Barcode** | **Sequence (5ʹ to 3ʹ)***^a^* |
| --- | --- |
| Btk1 | GAGCTCTGTCTCCAACTCCCAGTATCTTATTTTCTTAATAGATTATATCAAGACTATTAATTTTACCCGACGCAGGACCCCACTAAGTGATTTCAATATTTAGAAATTATTGTAAAATATCTTAACTTCGCTGCCTCAAACC**GCTAGC** |
| Btk2 | GAGCTCTCACACACCTATTTACTCCCTATTTTCAATTGTGTTTTAATTTAAGAGGATATATTACGCTCAAGGAAGCCTTCCCAAGTTCACACACTTATCTGTATATATAAATTTAAGAAAACAAAGGCGTGTACTAGACTTGCTT**GCTAGC** |
| Btk3 | GAGCTCAAACAGACGAGTCCTGAGAATCAATTATTTATCTAACTACAAACTATATTACAGTATTTACAACAACCAGAAAGACCCCTCCGGGAAATAATATTGTAAAATTAAAAGGTCTGAACAATATAGCGGAAGAGCAGTCTCTTAT**GCTAGC** |
| Btk4 | GAGCTCGTTGGTAGGCCAAACTACTTCTTACTTATAAGTTCGAAGATTTTAATATTGAATATCGATACAGGATCACGAGCGCCTCTAACTATAAACAATATACCTATTTCCAAAAGAAATAAAAATCGACAGTTATAGGAACTAGAC**GCTAGC** |
| Btk5 | GAGCTCCGATTTCTCAACCTATACACTGAGTGAGTTGTATTGTTAAAAAATCTCTTAAATAAACTCCGAGGTGATCACCAGAGTTCAGACTCAAGCTAATAAATCAACTTATATAAAATTAAGTCAGAAGTTCGGGTAATCAGAAGTT**GCTAGC** |
| Btk6 | GAGCTCGTCCGCCGCCCAATTTATATAAGTTTAAAAAAACTTAATAAAGATATTGTTCTAGGCCGCTTAAGCGCGCAGTTCTATTACTTTTTCTTTTTAAAATCAATATAATTTAGCCTAAACGCCGCTCAACGATTCTTTA**GCTAGC** |
| Btk7 | GAGCTCTGAGTGACAGGTGATCGACTTTATATTTATTATATTAGTAAGTCTTATCTACTATTAGCCTTCTAGTGAGTCGCCAACCGAGTTTTAACTGATTACTACTATAAATTTCAATTAATTTTTGGAGTAGTGCGCCGTTT**GCTAGC** |
| Btk8 | GAGCTCATTCTCACGGCTTCGACTTTCGTAAAAATAGTTTAGCTAAACTGAATTATTTTTAATTCGGTCCCCACTAATACCCCAGAGTGAGAAAGTTACACAATTTGAATTATATTTTAGTAATTTGCCTTAACTGACGGAACTCA**GCTAGC** |
| Btk9 | GAGCTCTCTGCCTGGAGTGATTTAGAAATATAGTTACAAACTATTTTAGAATATAATAGTGAGTACGTCGCTATATATCTCCCCACCCACCTATAAAGAATCCTAACAGTTTCTAGAAATAAAAATTAGTCTAATTCGTAGGCAACAG**GCTAGC** |
| Btk10 | GAGCTCTAATATCGGCAGACGTGCTGTTTACTGAAATTAAACTTATTAGAACTAAATACTACTTACCGTTAAGCTACAGGACACCGCAACGAAAAATCACGATAATCTATATAATTACCGTATAAAAACTGAATCAAACTGGGTGATCT**GCTAGC** |
| Btk11 | GAGCTCACGGAGACGGTCTGTTATTGTAATCAAAAGACTAAACAAAAATTTCTAAAATTAAAGCGGCACTGCGCTAAACTCCCTTAGATATATTTTGCTTATAATAAAATACTAAGTACAATCTCGGCTATCCCTATTCAGT**GCTAGC** |
| Btk12 | GAGCTCCGTCGGTATTGCACCTTACAACGATTAATTTATAATAATTAAGTTGTATTATCTCTGAAGCGTCCTCTAGTCCACTTGGAGCAAAATTATTATCACTTATCTAATCTTAAATTTTACACGTGGATCACAACGCTAT**GCTAGC** |
| Bp1 | GCCTCACTTGAGGTAATTAGAATCTGCGGGGCGCGCGGGCGAACCCGACGGCGGGGGATCGCGACTCACTCCTACACGTAAAAGGGCTGCCCGTCGCTCCCGCCCGGGGGGGCTGCCGGGGATTGCCCCTTCGTTAAACCA |
| Bp2 | ATATAGCCGCGGACAGATCTTACGGCCGAGTCCGCCCCGGCAGCGCCCCCGCCTAGGGAGTCCGACTTTCAAATCACCGCACCCAGCCCGGCCGCCGCTAGCTCGGGAGGGCGGTGTACCAAGCCCGGTTGTTTCA |
| Bp3 | AGGCAGGTGGCGAGTAAATAGGTCTCGCTCGTCGCTGGGACCGCCCCGGGTGGGCTGCCGATAGGCCCTTACAACTCTGCGGCCGCCCGTGCTGGCTGGCAGCAGTCGACCCGGCCCTGGACACGCTCAAGGTTAT |
| Cbot1 | GTGGTGCGAGACTGTAAAGATAATTAAAAATATTAATTTAGTTTATTTTTAAATAAGGGTCACACGTTGCCCTTCTTCTATCGTAATTAAATTTAAATTAATAATAAATTTTAAGTAACGACGTTGTTTAGGGTTACG |
| Cbot2 | TTCGCTCTGCAAATTTGAAATCGTTATTAGATTAAAATTACTTTATTAAAAAAATCTTATTGCCCAAGGATACGTGCACCTCTAGGAAAATTTTTAAGATTATATTTTAATAGTATTTTAGAAAGCAGACGAAAGATTGACTAC |
| Cbot3 | CGGAATAGTGAACGGTTCCTTTTAATTATTTAAATTATAAAAAAAATAATCAATATTGAAGCGTCGTCCTAGCCCTTATTGCAGATTTCTTTAAAATAAAATAATTATATAATTATAAAAGGAGTCAGTAGCTGTTGTGA |
| Yp1 | AAGATTACGAGTTGGCACGAAATTCGAGTATTATAGGTCTAGCTCTGCGAGAAGACCGCTTAAGCTCGGGTACCTATCCACCGAGTACCCGCGCTCTTAGCTAGATTAGTTCTTACACCCTTCGTAAAGTTACCGGTTGAAGA |
| Yp2 | TGTTGTCGGAGACCTATTCTGTTTTCTGTGAGGGACTAAGAATAGCCTTACTCCTGCCGTACCCGAAGTGACGTACTTCTCCTGAGCCTCCGAGTATTATTGGATCACCGCAACTGTTTGGACAGAGACTGGTTAATTATTG |
| Yp3 | CCGTAGCTGTTGATCGTCAAAAATCACGATTCTTTGGTACGAGGTCAATCACCACACCAGGTGGCCCAAATCTTATACGGAACCTCGGCTTTGTAGAACGATTCCTAACGTGAAGTCAGGCAACTTGCTAAGAAGTCGAA |

*^a^*SacI restriction sites are underlined; NheI restriction sites are shown in bold.

Table S2. Barcode primer and probe sequences.

| **Barcode** | **Forward Primer (5ʹ to 3ʹ)** |
| --- | --- |
| Btk1 | TGTCTCCAACTCCCAGTATC |
| Btk2 | TCACACACCTATTTACTCCCTAT |
| Btk3 | AAACAGACGAGTCCTGAGAATC |
| Btk4 | GTTGGTAGGCCAAACTACTTC |
| Btk5 | CGATTTCTCAACCTATACACTGA |
| Btk6 | GTCCGCCGCCCAATTTATA |
| Btk7 | TGAGTGACAGGTGATCGACTT |
| Btk8 | ATTCTCACGGCTTCGACTTTC |
| Btk9 | TCTGCCTGGAGTGATTTAGAAAT |
| Btk10 | TAATATCGGCAGACGTGCTGTT |
| Btk11 | ACGGAGACGGTCTGTTATTG |
| Btk12 | CGTCGGTATTGCACCTTAC |
| Bp1 | GCCTCACTTGAGGTAATTAGAAT |
| Bp2 | ATATAGCCGCGGACAGATC |
| Bp3 | AGGCAGGTGGCGAGTAAATA |
| Cbot1 | GTGGTGCGAGACTGTAAAGAT |
| Cbot2 | TTCGCTCTGCAAATTTGAAATCG |
| Cbot3 | CGGAATAGTGAACGGTTCCTTT |
| Yp1 | AAGATTACGAGTTGGCACGAAAT |
| Yp2 | TGTTGTCGGAGACCTATTCTGT |
| Yp3 | CCGTAGCTGTTGATCGTCAAA |
|  | **Reverse Primer (5ʹ to 3ʹ)** |
| Btk1 | GGTTTGAGGCAGCGAAGTTA |
| Btk2 | AAGCAAGTCTAGTACACGCC |
| Btk3 | ATAAGAGACTGCTCTTCCGCTA |
| Btk4 | GTCTAGTTCCTATAACTGTCGAT |
| Btk5 | AACTTCTGATTACCCGAACTTCT |
| Btk6 | TAAAGAATCGTTGAGCGGCGT |
| Btk7 | AAACGGCGCACTACTCCAAA |
| Btk8 | TGAGTTCCGTCAGTTAAGGCA |
| Btk9 | CTGTTGCCTACGAATTAGACTAA |
| Btk10 | AGATCACCCAGTTTGATTCAGTT |
| Btk11 | ACTGAATAGGGATAGCCGAG |
| Btk12 | ATAGCGTTGTGATCCACGTG |
| Bp1 | TGGTTTAACGAAGGGGCAATC |
| Bp2 | TGAAACAACCGGGCTTGGTA |
| Bp3 | ATAACCTTGAGCGTGTCCAG |
| Cbot1 | CGTAACCCTAAACAACGTCG |
| Cbot2 | GTAGTCAATCTTTCGTCTGCTTT |
| Cbot3 | TCACAACAGCTACTGACTCCTT |
| Yp1 | TCTTCAACCGGTAACTTTACGAA |
| Yp2 | CAATAATTAACCAGTCTCTGTCC |
| Yp3 | TTCGACTTCTTAGCAAGTTGCC |
|  | **Probe (5ʹ to 3ʹ)** |
| Btk1 | TTTACCCGACGCAGGACCCCACTAAG |
| Btk2 | CGCTCAAGGAAGCCTTCCCAAGTTCACA |
| Btk3 | TTACAACAACCAGAAAGACCCCTCCGGG |
| Btk4 | CGATACAGGATCACGAGCGCCTCTAAC |
| Btk5 | CTCCGAGGTGATCACCAGAGTTCAGACT |
| Btk6 | AGGCCGCTTAAGCGCGCAGTTCTATTAC |
| Btk7 | TAGCCTTCTAGTGAGTCGCCAACCGAGT |
| Btk8 | TTCGGTCCCCACTAATACCCCAGAGTGA |
| Btk9 | TACGTCGCTATATATCTCCCCACCCACC |
| Btk10 | TACCGTTAAGCTACAGGACACCGCAACG |
| Btk11 | AGCGGCACTGCGCTAAACTCCCTTAG |
| Btk12 | CTGAAGCGTCCTCTAGTCCACTTGGAG |
| Bp1 | ATCGCGACTCACTCCTACACGTAAAAGGG |
| Bp2 | TAGGGAGTCCGACTTTCAAATCACCGCAC |
| Bp3 | TGCCGATAGGCCCTTACAACTCTGCG |
| Cbot1 | AGGGTCACACGTTGCCCTTCTTCTATCGT |
| Cbot2 | ATTGCCCAAGGATACGTGCACCTCTAGG |
| Cbot3 | TGAAGCGTCGTCCTAGCCCTTATTGCAG |
| Yp1 | CTTAAGCTCGGGTACCTATCCACCGAG |
| Yp2 | CGTACCCGAAGTGACGTACTTCTCCTG |
| Yp3 | ACCAGGTGGCCCAAATCTTATACGGAACC |

Table S3. Constraints of properties of sequences tested experimentally in this study. Note that all instances where sequences do not match the default have been traced to errors in the code that have since been corrected.

| **Barcode** | **Type** | **Length (bases)** | **T_m_*^a^***  **(**°**C)** | **G+C content (%)** | **#C’s ≥ #G’s** |
| --- | --- | --- | --- | --- | --- |
|  |  |  |  |  |  |
| *Default (primers)* | *Min:* | *20* | *58* | *40* | *n/a* |
|  | *Max:* | *24* | *60* | *60* |  |
| *Default (probes)* | *Min:* | *20* | *68* | *40* | *True* |
|  | *Max:* | *30* | *70* | *60* |  |
|  |  |  |  |  |  |
| Btk1 | F Primer | 20 | 56.3 | 50.0 | n/a |
| Btk2 | F Primer | 23 | 56.2 | 39.1 | n/a |
| Btk3 | F Primer | 22 | 57.5 | 45.5 | n/a |
| Btk4 | F Primer | 21 | 56.9 | 47.6 | n/a |
| Btk5 | F Primer | 23 | 56.2 | 39.1 | n/a |
| Btk6 | F Primer | 19 | 55.6 | 52.6 | n/a |
| Btk7 | F Primer | 21 | 56.9 | 47.6 | n/a |
| Btk8 | F Primer | 21 | 56.9 | 47.6 | n/a |
| Btk9 | F Primer | 23 | 56.2 | 39.1 | n/a |
| Btk10 | F Primer | 22 | 57.5 | 45.5 | n/a |
| Btk11 | F Primer | 20 | 56.3 | 50.0 | n/a |
| Btk12 | F Primer | 19 | 55.6 | 52.6 | n/a |
| Bp1 | F Primer | 23 | 56.2 | 39.1 | n/a |
| Bp2 | F Primer | 19 | 55.6 | 52.6 | n/a |
| Bp3 | F Primer | 20 | 56.3 | 50.0 | n/a |
| Cbot1 | F Primer | 21 | 56.9 | 47.6 | n/a |
| Cbot2 | F Primer | 23 | 56.2 | 39.1 | n/a |
| Cbot3 | F Primer | 22 | 57.5 | 45.5 | n/a |
| Yp1 | F Primer | 23 | 56.2 | 39.1 | n/a |
| Yp2 | F Primer | 22 | 57.5 | 45.5 | n/a |
| Yp3 | F Primer | 21 | 56.9 | 47.6 | n/a |
| Btk1 | R Primer | 20 | 56.3 | 50.0 | n/a |
| Btk2 | R Primer | 20 | 56.3 | 50.0 | n/a |
| Btk3 | R Primer | 22 | 57.5 | 45.5 | n/a |
| Btk4 | R Primer | 23 | 56.2 | 39.1 | n/a |
| Btk5 | R Primer | 23 | 56.2 | 39.1 | n/a |
| Btk6 | R Primer | 21 | 56.9 | 47.6 | n/a |
| Btk7 | R Primer | 20 | 56.3 | 50.0 | n/a |
| Btk8 | R Primer | 21 | 56.9 | 47.6 | n/a |
| Btk9 | R Primer | 23 | 56.2 | 39.1 | n/a |
| Btk10 | R Primer | 23 | 56.2 | 39.1 | n/a |
| Btk11 | R Primer | 20 | 56.3 | 50.0 | n/a |
| Btk12 | R Primer | 20 | 56.3 | 50.0 | n/a |
| Bp1 | R Primer | 21 | 56.9 | 47.6 | n/a |
| Bp2 | R Primer | 20 | 56.3 | 50.0 | n/a |
| Bp3 | R Primer | 20 | 56.3 | 50.0 | n/a |
| Cbot1 | R Primer | 20 | 56.3 | 50.0 | n/a |
| Cbot2 | R Primer | 23 | 56.2 | 39.1 | n/a |
| Cbot3 | R Primer | 22 | 57.5 | 45.5 | n/a |
| Yp1 | R Primer | 23 | 56.2 | 39.1 | n/a |
| Yp2 | R Primer | 23 | 56.2 | 39.1 | n/a |
| Yp3 | R Primer | 22 | 57.5 | 45.5 | n/a |
| Btk1 | Probe | 26 | 67.2 | 57.7 | True |
| Btk2 | Probe | 28 | 67.4 | 53.6 | True |
| Btk3 | Probe | 28 | 67.4 | 53.6 | True |
| Btk4 | Probe | 27 | 67.3 | 55.6 | True |
| Btk5 | Probe | 28 | 67.4 | 53.6 | True |
| Btk6 | Probe | 28 | 67.4 | 53.6 | True |
| Btk7 | Probe | 28 | 67.4 | 53.6 | True |
| Btk8 | Probe | 28 | 67.4 | 53.6 | True |
| Btk9 | Probe | 28 | 67.4 | 53.6 | True |
| Btk10 | Probe | 28 | 67.4 | 53.6 | True |
| Btk11 | Probe | 26 | 67.2 | 57.7 | True |
| Btk12 | Probe | 27 | 67.3 | 55.6 | True |
| Bp1 | Probe | 29 | 67.4 | 51.7 | True |
| Bp2 | Probe | 29 | 67.4 | 51.7 | True |
| Bp3 | Probe | 26 | 67.2 | 57.7 | True |
| Cbot1 | Probe | 29 | 67.4 | 51.7 | True |
| Cbot2 | Probe | 28 | 67.4 | 53.6 | True |
| Cbot3 | Probe | 28 | 67.4 | 53.6 | True |
| Yp1 | Probe | 27 | 67.3 | 55.6 | True |
| Yp2 | Probe | 27 | 67.3 | 55.6 | True |
| Yp3 | Probe | 29 | 67.4 | 51.7 | True |

*^a^*Computed using Equation (1) in main text.

Table S4. Checks of properties of sequences tested experimentally in this study. Note that all instances where sequences do not match the default have been traced to errors in the code that have since been corrected.

| **Barcode** | **Type** | **Max repeats (G)** | **Max repeats**  **(A/T/C)** | **No G on 5ʹ end** | **G/C’s in last 5 bases of 3ʹ end** | **No start codons present** | **No stem-loop detected** |
| --- | --- | --- | --- | --- | --- | --- | --- |
|  |  |  |  |  |  |  |  |
| *Default (primers)* |  | *3* | *4* | *n/a* | *2* | *True* | *See Table 1* |
| *Default (probes)* |  | *3* | *4* | *True* | *n/a* | *True* | *See Table 1* |
|  |  |  |  |  |  |  |  |
| Btk1 | F Primer | 3 | 1 | n/a | 2 | True | True |
| Btk2 | F Primer | 3 | 1 | n/a | 2 | True | True |
| Btk3 | F Primer | 3 | 1 | n/a | 2 | True | True |
| Btk4 | F Primer | 3 | 2 | n/a | 2 | True | True |
| Btk5 | F Primer | 3 | 1 | n/a | 2 | True | True |
| Btk6 | F Primer | 3 | 1 | n/a | 0 | True | True |
| Btk7 | F Primer | 2 | 2 | n/a | 2 | True | True |
| Btk8 | F Primer | 3 | 2 | n/a | 2 | True | True |
| Btk9 | F Primer | 3 | 2 | n/a | 1 | True | True |
| Btk10 | F Primer | 2 | 2 | n/a | 2 | True | True |
| Btk11 | F Primer | 2 | 2 | n/a | 1 | True | True |
| Btk12 | F Primer | 2 | 2 | n/a | 2 | True | True |
| Bp1 | F Primer | 2 | 2 | n/a | 1 | True | True |
| Bp2 | F Primer | 2 | 2 | n/a | 2 | True | True |
| Bp3 | F Primer | 3 | 2 | n/a | 0 | True | True |
| Cbot1 | F Primer | 3 | 2 | n/a | 1 | True | True |
| Cbot2 | F Primer | 3 | 1 | n/a | 2 | True | True |
| Cbot3 | F Primer | 3 | 2 | n/a | 2 | True | True |
| Yp1 | F Primer | 3 | 2 | n/a | 1 | True | True |
| Yp2 | F Primer | 2 | 2 | n/a | 2 | True | True |
| Yp3 | F Primer | 3 | 1 | n/a | 1 | True | True |
| Btk1 | R Primer | 3 | 2 | n/a | 1 | True | True |
| Btk2 | R Primer | 2 | 1 | n/a | 4 | True | True |
| Btk3 | R Primer | 2 | 1 | n/a | 3 | True | True |
| Btk4 | R Primer | 2 | 1 | n/a | 2 | True | True |
| Btk5 | R Primer | 3 | 1 | n/a | 2 | True | True |
| Btk6 | R Primer | 3 | 2 | n/a | 4 | True | True |
| Btk7 | R Primer | 3 | 2 | n/a | 2 | True | True |
| Btk8 | R Primer | 2 | 2 | n/a | 3 | True | True |
| Btk9 | R Primer | 2 | 1 | n/a | 1 | True | True |
| Btk10 | R Primer | 3 | 1 | n/a | 2 | True | True |
| Btk11 | R Primer | 2 | 3 | n/a | 4 | True | True |
| Btk12 | R Primer | 2 | 1 | n/a | 3 | True | True |
| Bp1 | R Primer | 3 | 4 | n/a | 2 | True | True |
| Bp2 | R Primer | 3 | 3 | n/a | 2 | True | True |
| Bp3 | R Primer | 2 | 1 | n/a | 3 | True | True |
| Cbot1 | R Primer | 3 | 1 | n/a | 4 | True | True |
| Cbot2 | R Primer | 3 | 1 | n/a | 2 | True | True |
| Cbot3 | R Primer | 2 | 1 | n/a | 2 | True | True |
| Yp1 | R Primer | 3 | 2 | n/a | 2 | True | True |
| Yp2 | R Primer | 2 | 1 | n/a | 3 | True | True |
| Yp3 | R Primer | 2 | 1 | n/a | 3 | True | False |
| Btk1 | Probe | 4 | 2 | True | n/a | True | True |
| Btk2 | Probe | 3 | 2 | True | n/a | True | True |
| Btk3 | Probe | 4 | 3 | True | n/a | True | True |
| Btk4 | Probe | 2 | 2 | True | n/a | True | True |
| Btk5 | Probe | 2 | 2 | True | n/a | True | True |
| Btk6 | Probe | 2 | 2 | True | n/a | True | True |
| Btk7 | Probe | 2 | 1 | True | n/a | True | True |
| Btk8 | Probe | 4 | 2 | True | n/a | True | True |
| Btk9 | Probe | 4 | 1 | True | n/a | True | True |
| Btk10 | Probe | 2 | 2 | True | n/a | True | True |
| Btk11 | Probe | 3 | 2 | True | n/a | True | True |
| Btk12 | Probe | 2 | 2 | True | n/a | True | True |
| Bp1 | Probe | 4 | 3 | True | n/a | True | True |
| Bp2 | Probe | 3 | 3 | True | n/a | True | True |
| Bp3 | Probe | 3 | 2 | True | n/a | True | True |
| Cbot1 | Probe | 3 | 3 | True | n/a | True | True |
| Cbot2 | Probe | 3 | 2 | True | n/a | True | True |
| Cbot3 | Probe | 3 | 1 | True | n/a | True | True |
| Yp1 | Probe | 2 | 3 | True | n/a | True | True |
| Yp2 | Probe | 3 | 1 | True | n/a | True | True |
| Yp3 | Probe | 3 | 2 | True | n/a | True | True |

Table S5. BLAST results for sequences tested experimentally in this study. Btk, Bp, Cbot, and Yp are shorthand for *B. thuringiensis* serovar*kurstaki,* *B. pseudomallei*1026b, *C. botulinum* Hall A, and *Y. pestis* CO92, respectively (see Methods for more information).

| **Barcode** | **Type** | **Max BLAST Hit Scores*^a^*** | | | | | |
| --- | --- | --- | --- | --- | --- | --- | --- |
|  |  | **Assays in this Study** | **Btk** | **Bp** | **Cbot** | **Yp** | **NCBI Database*^b^*** |
|  |  |  |  |  |  |  |  |
| *Default (primers)* |  | *0.85* | *0.85* | *0.85* | *0.85* | *0.85* | *0.85* |
| *Default (probes)* |  | *0.85* | *0.85* | *0.85* | *0.85* | *0.85* | *0.85* |
|  |  |  |  |  |  |  |  |
| Btk1 | F Primer | 0.35 | 0.65 | 0.55 | 0.65 | 0.65 | 0.90 |
| Btk2 | F Primer | 0.39 | 0.00 | 0.65 | 0.57 | 0.57 | 0.87 |
| Btk3 | F Primer | 0.32 | 0.50 | 0.55 | 0.55 | 0.55 | 0.86 |
| Btk4 | F Primer | 0.00 | 0.52 | 0.57 | 0.62 | 0.57 | 0.00 |
| Btk5 | F Primer | 0.30 | 0.00 | 0.57 | 0.52 | 0.52 | 0.83 |
| Btk6 | F Primer | 0.00 | 0.68 | 0.63 | 0.63 | 0.68 | 0.00 |
| Btk7 | F Primer | 0.38 | 0.67 | 0.57 | 0.62 | 0.67 | 0.86 |
| Btk8 | F Primer | 0.38 | 0.71 | 0.57 | 0.00 | 0.62 | 0.86 |
| Btk9 | F Primer | 0.30 | 0.57 | 0.57 | 0.57 | 0.70 | 0.83 |
| Btk10 | F Primer | 0.36 | 0.59 | 0.55 | 0.50 | 0.59 | 0.86 |
| Btk11 | F Primer | 0.35 | 0.65 | 0.60 | 0.55 | 0.60 | 0.90 |
| Btk12 | F Primer | 0.37 | 0.63 | 0.63 | 0.58 | 0.63 | 0.95 |
| Bp1 | F Primer | 0.30 | 0.48 | 0.52 | 0.52 | 0.57 | 0.83 |
| Bp2 | F Primer | 0.37 | 0.68 | 0.58 | 0.00 | 0.68 | 0.00 |
| Bp3 | F Primer | 0.45 | 0.75 | 0.60 | 0.55 | 0.60 | 0.00 |
| Cbot1 | F Primer | 0.33 | 0.52 | 0.62 | 0.52 | 0.57 | 0.86 |
| Cbot2 | F Primer | 0.35 | 0.52 | 0.61 | 0.52 | 0.61 | 0.78 |
| Cbot3 | F Primer | 0.00 | 0.50 | 0.55 | 0.55 | 0.59 | 0.82 |
| Yp1 | F Primer | 0.30 | 0.48 | 0.52 | 0.52 | 0.57 | 0.78 |
| Yp2 | F Primer | 0.32 | 0.55 | 0.55 | 0.55 | 0.50 | 0.82 |
| Yp3 | F Primer | 0.48 | 0.71 | 0.62 | 0.52 | 0.62 | 0.90 |
| Btk1 | R Primer | 0.00 | 0.65 | 0.70 | 0.55 | 0.65 | 0.90 |
| Btk2 | R Primer | 0.35 | 0.55 | 0.60 | 0.65 | 0.55 | 0.90 |
| Btk3 | R Primer | 0.36 | 0.55 | 0.59 | 0.59 | 0.55 | 0.86 |
| Btk4 | R Primer | 0.30 | 0.00 | 0.61 | 0.57 | 0.57 | 0.78 |
| Btk5 | R Primer | 0.35 | 0.65 | 0.52 | 0.57 | 0.65 | 0.83 |
| Btk6 | R Primer | 0.33 | 0.62 | 0.57 | 0.57 | 0.62 | 0.86 |
| Btk7 | R Primer | 0.00 | 0.65 | 0.60 | 0.60 | 0.65 | 0.90 |
| Btk8 | R Primer | 0.33 | 0.62 | 0.67 | 0.62 | 0.67 | 0.86 |
| Btk9 | R Primer | 0.30 | 0.52 | 0.52 | 0.52 | 0.57 | 0.78 |
| Btk10 | R Primer | 0.30 | 0.52 | 0.52 | 0.57 | 0.61 | 0.83 |
| Btk11 | R Primer | 0.35 | 0.65 | 0.60 | 0.65 | 0.70 | 0.90 |
| Btk12 | R Primer | 0.35 | 0.60 | 0.70 | 0.55 | 0.60 | 0.90 |
| Bp1 | R Primer | 0.33 | 0.62 | 0.62 | 0.52 | 0.62 | 0.86 |
| Bp2 | R Primer | 0.35 | 0.60 | 0.65 | 0.55 | 0.65 | 0.00 |
| Bp3 | R Primer | 0.45 | 0.65 | 0.55 | 0.60 | 0.75 | 0.90 |
| Cbot1 | R Primer | 0.35 | 0.55 | 0.60 | 0.65 | 0.60 | 0.00 |
| Cbot2 | R Primer | 0.30 | 0.57 | 0.57 | 0.57 | 0.57 | 0.87 |
| Cbot3 | R Primer | 0.45 | 0.55 | 0.64 | 0.50 | 0.59 | 0.86 |
| Yp1 | R Primer | 0.30 | 0.52 | 0.65 | 0.52 | 0.57 | 0.78 |
| Yp2 | R Primer | 0.35 | 0.48 | 0.65 | 0.52 | 0.57 | 0.83 |
| Yp3 | R Primer | 0.36 | 0.50 | 0.64 | 0.55 | 0.59 | 0.86 |
| Btk1 | Probe | 0.35 | 0.54 | 0.50 | 0.00 | 0.46 | 0.73 |
| Btk2 | Probe | 0.32 | 0.46 | 0.43 | 0.50 | 0.43 | 0.68 |
| Btk3 | Probe | 0.25 | 0.46 | 0.50 | 0.46 | 0.43 | 0.71 |
| Btk4 | Probe | 0.26 | 0.56 | 0.52 | 0.44 | 0.48 | 0.70 |
| Btk5 | Probe | 0.29 | 0.46 | 0.46 | 0.00 | 0.50 | 0.68 |
| Btk6 | Probe | 0.25 | 0.50 | 0.43 | 0.43 | 0.46 | 0.00 |
| Btk7 | Probe | 0.36 | 0.39 | 0.43 | 0.43 | 0.46 | 0.68 |
| Btk8 | Probe | 0.32 | 0.43 | 0.43 | 0.43 | 0.46 | 0.68 |
| Btk9 | Probe | 0.25 | 0.39 | 0.46 | 0.43 | 0.50 | 0.71 |
| Btk10 | Probe | 0.25 | 0.43 | 0.46 | 0.43 | 0.46 | 0.00 |
| Btk11 | Probe | 0.27 | 0.46 | 0.00 | 0.42 | 0.46 | 0.00 |
| Btk12 | Probe | 0.33 | 0.48 | 0.48 | 0.48 | 0.52 | 0.70 |
| Bp1 | Probe | 0.34 | 0.41 | 0.45 | 0.45 | 0.48 | 0.66 |
| Bp2 | Probe | 0.28 | 0.45 | 0.48 | 0.45 | 0.45 | 0.66 |
| Bp3 | Probe | 0.31 | 0.50 | 0.46 | 0.46 | 0.50 | 0.77 |
| Cbot1 | Probe | 0.00 | 0.48 | 0.55 | 0.41 | 0.48 | 0.66 |
| Cbot2 | Probe | 0.25 | 0.43 | 0.43 | 0.46 | 0.43 | 0.68 |
| Cbot3 | Probe | 0.32 | 0.39 | 0.46 | 0.43 | 0.43 | 0.00 |
| Yp1 | Probe | 0.30 | 0.44 | 0.00 | 0.00 | 0.52 | 0.00 |
| Yp2 | Probe | 0.30 | 0.52 | 0.52 | 0.44 | 0.44 | 0.70 |
| Yp3 | Probe | 0.28 | 0.45 | 0.41 | 0.41 | 0.41 | 0.66 |

*^a^*Scores of 0.00 correspond to no BLAST hits returned.

*^b^*Checks against the NCBI database presented here were made using a more current image of the NCBI database than when the sequences were generated; hits above the target threshold are presumed to be due to expansion of the database.

Table S6. Checks of properties of barcodes tested experimentally in this study. Note that all instances where sequences do not match the default have been traced to errors in the code that have since been corrected.

| **Barcode** | **Spacer size (bases)** | **No stem-loop detected** | **Barcode G+C content*^a^* (%)** | **Target organism G+C content (%)** |
| --- | --- | --- | --- | --- |
|  |  |  |  |  |
| *Default* | *100* | *See Table 2* | *See ‘Target organism G+C content’ column* | *n/a* |
|  |  |  |  |  |
| Btk1 | 96 | True | 33.1 | 35.3 |
| Btk2 | 96 | True | 33.1 |  |
| Btk3 | 98 | True | 33.1 |  |
| Btk4 | 97 | True | 32.6 |  |
| Btk5 | 96 | True | 33.1 |  |
| Btk6 | 96 | True | 33.1 |  |
| Btk7 | 96 | True | 32.9 |  |
| Btk8 | 98 | True | 33.6 |  |
| Btk9 | 96 | True | 33.1 |  |
| Btk10 | 98 | True | 33.6 |  |
| Btk11 | 96 | True | 33.1 |  |
| Btk12 | 97 | True | 33.1 |  |
| Bp1 | 97 | True | 66.7 | 68.2 |
| Bp2 | 97 | True | 66.9 |  |
| Bp3 | 96 | True | 66.9 |  |
| Cbot1 | 97 | True | 26.8 | 28.2 |
| Cbot2 | 98 | True | 27.1 |  |
| Cbot3 | 96 | True | 26.4 |  |
| Yp1 | 97 | True | 46.9 | 47.6 |
| Yp2 | 97 | True | 46.5 |  |
| Yp3 | 97 | True | 46.4 |  |

*^a^*G+C content of barcodes generated by the algorithm, not inclusive of restriction sites added to ends of Btk barcodes for cloning.

Table S7. Sources of DNA for cross-reactivity panel.

| **DNA Template** | **Source** | **Catalog #** |
| --- | --- | --- |
| *Bacillus anthracis* Ames35 | BEI*^a^* | NR-10450 |
| *Bacillus anthracis* Sterne 34F2 | S. Leppla (NIH)*^b^* | − |
| *Bacillus cereus* Gibson 971 | BEI*^a^* | NR-4198 |
| *Bacillus licheniformis* Gibson 46 | BEI*^a^* | NR-2546 |
| *Bacillus megaterium* Ford 19 | BEI*^a^* | NR-2543 |
| *Bacillus sphaericus* Ford 25 | BEI*^a^* | NR-2548 |
| *Bacillus subtilis* subsp. *subtilis* 168 | BGSC*^b^* | 1A1 |
| *Bacillus thuringiensis* serovar *kurstaki* HD-1 | BEI*^b^* | NR-610 |
| *Bacillus thuringiensis* subsp. *konkukian* 97-27 | BEI*^a^* | NR-12315 |
| *Burkholderia pseudomallei* 1026b | BEI*^a^* | NR-9321 |
| *Clostridium perfringens* WAL-14572 | BEI*^a^* | HM-310D |
| *Escherichia coli* EDL933 | BEI*^a^* | NR-2648 |
| *Francisella tularensis* subsp. *tularensis* SCHU S4 | BEI*^a^* | NR-3015 |
| Microbial Mock Community B (Even, High Concentration) | BEI*^a^* | HM-276D |
| *Micrococcus luteus* SK58 | BEI*^a^* | HM-114D |
| *Neisseria meningitidis* 9506 | BEI*^a^* | NR-48805 |
| *Pseudomonas* sp. 2_1_26 | BEI*^a^* | HM-214D |
| *Salmonella enterica* subsp. *enterica* LT2 | BEI*^a^* | NR-4218 |
| Soil DNA extract | Abingdon, MD*^c^* | − |
| *Staphylococcus aureus* TCH1516 | BEI*^a^* | NR-12252 |
| *Staphylococcus epidermidis* SK135 | BEI*^a^* | HM-118D |
| *Streptococcus pneumoniae* TCH8431 | BEI*^a^* | HM-145D |
| *Vibrio cholerae* 395 | BEI*^a^* | NR-15694 |
| *Yersinia pestis* CO92 | BEI*^a^* | NR-2717 |

*^a^*Genomic DNA was obtained from the indicated commercial source; BEI, BEI Resources (Manassas, VA).

*^b^*Organism was obtained from the indicated source and genomic DNA extracted in-house using UltraClean Microbial DNA Isolation Kit (MOBIO Laboratories, Inc., Carlsbad, CA); BGSC, Bacillus Genetic Stock Center (Columbus, OH).

*^c^*Sample was collected at the indicated location and genomic DNA extracted in-house using DNeasy PowerSoil Kit (Qiagen, Hilden, Germany).

Table S8. Primers for constructing barcoded *Y. pestis* strain.

| **Primer #** | **Primer Name** | **Sequence (5ʹ→3ʹ)***^a^* |
| --- | --- | --- |
| 1 | pKD3_Cm-F | CTA**GAGCTC**TGTGACGGAAGATCAC |
| 2 | pKD3_Cm_SacB-R | CTATTAGACTCGAATAGGAACTTCGGAATAGG |
| 3 | p88171_SacB_Cm-F | CGAAGTTCCTATTCGAGTCTAATAGAATGAGGTCG |
| 4 | p88171_SacB-R | CAT**GGATCC**TGCCAATAGGATATCGGC |
| 5 | Cm_Yp2-F | *CAATCTACATCAACTTAC*CTGTGACGGAAGATCAC |
| 6 | SacB_Yp2-R | *GATGTACCTACATCA*TGCCAATAGGATATCGGC |
| 7 | Yp2_UpFlank_F | *GGATAGGGGATATGCTGC* |
| 8 | Yp2_UpFlank_Cm-R | GTCACAG*GTAAGTTGATGTAGATTGAATCAG* |
| 9 | Yp2_DownFlank_SacB-F | CCTATTGGCA*TGATGTAGGTACATCTGCGG* |
| 10 | Yp2_DownFlank_R | *CGTGAACTCTGCCGCAAG* |
| 11 | Yp2_Barcode1_F | *ACATCAACTTAC*AAGATTACGAGTTGGCAC |
| 12 | Yp2_Barcode1_R | *CCTACATCA*TCTTCAACCGGTAACTTTAC |
| 13 | Yp2_UpFlank_R | CTCGTAATCTT*GTAAGTTGATGTAGATTGAATCAG* |
| 14 | Yp2_DownFlank_F | CGGTTGAAGA*TGATGTAGGTACATCTGCGG* |

*^a^*Restriction sites are in bold. Bases in italics represent regions that align with the *Y. pestis* CO92 *pgm*^−^ chromosome. Underlined sequences align with barcode Yp1.


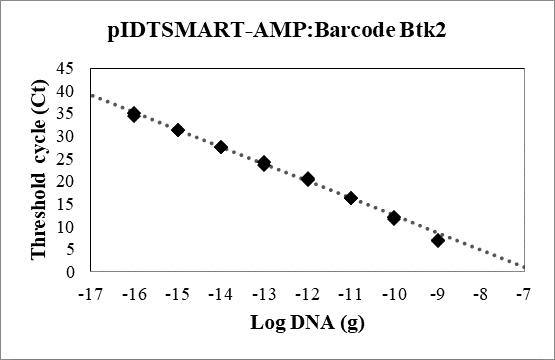

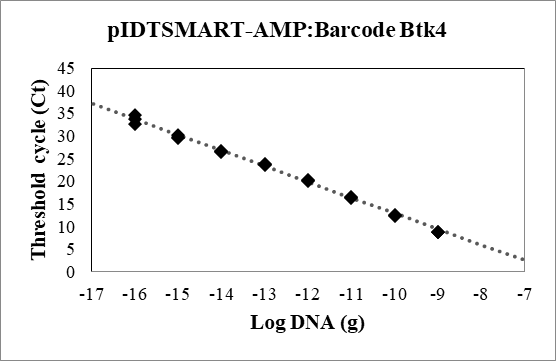

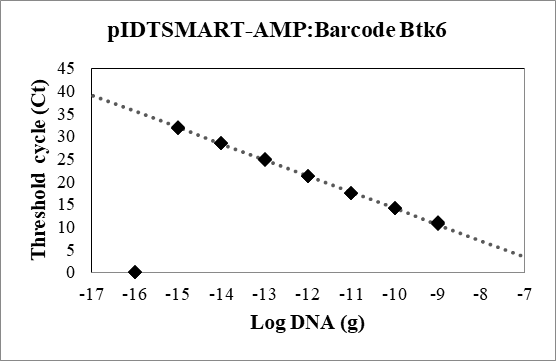

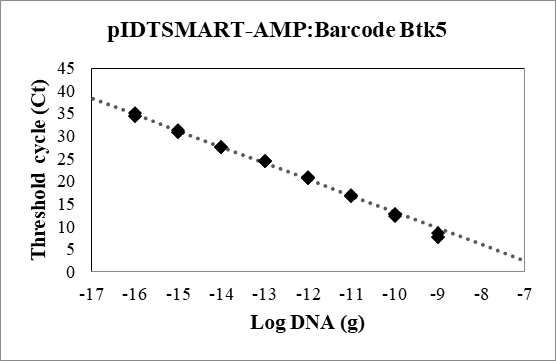

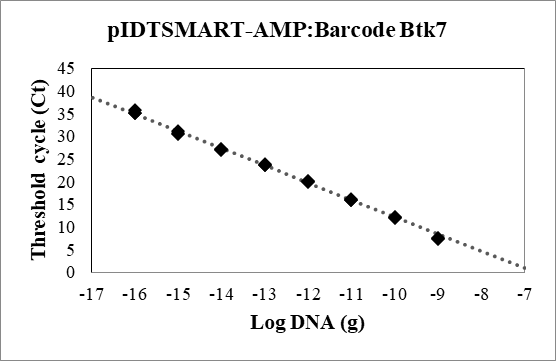

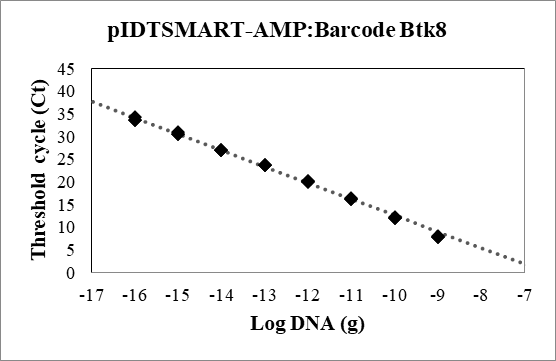

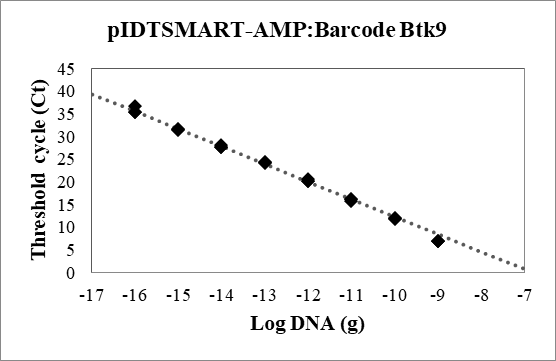

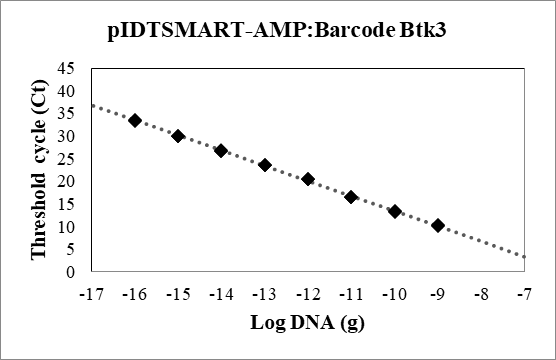


⁂


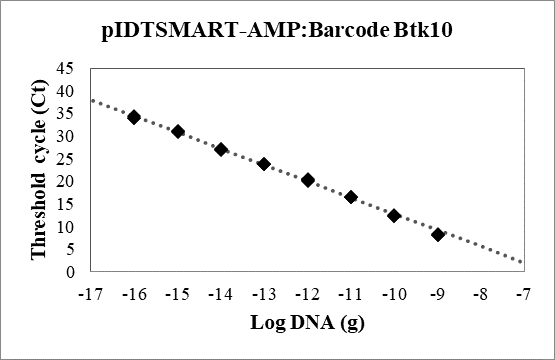

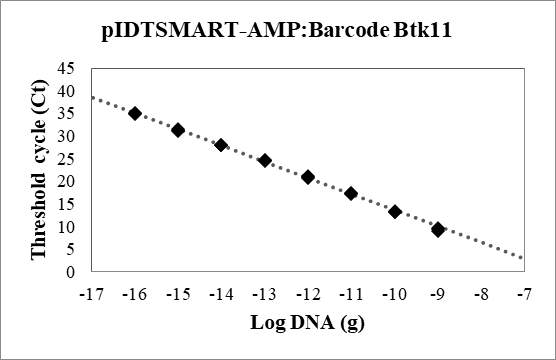

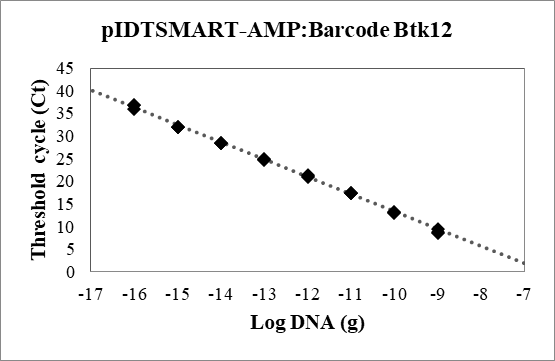

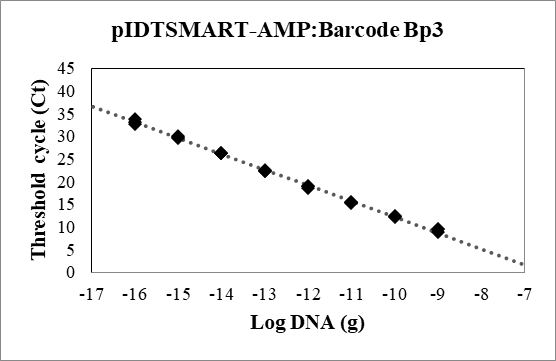

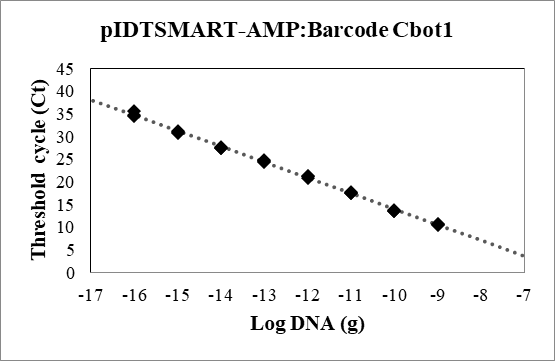

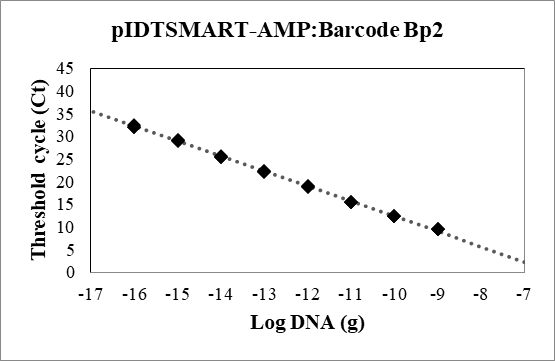

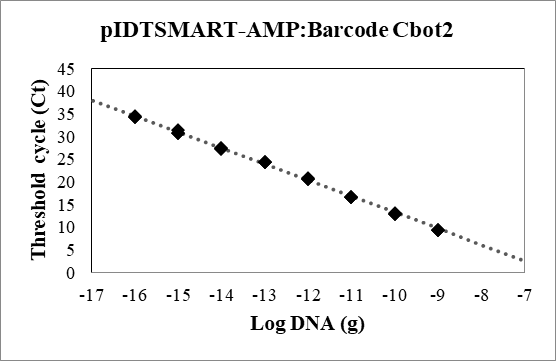

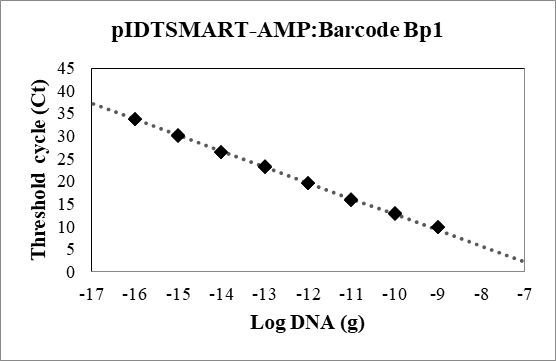

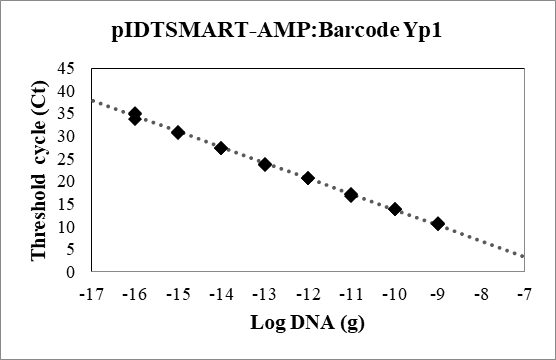

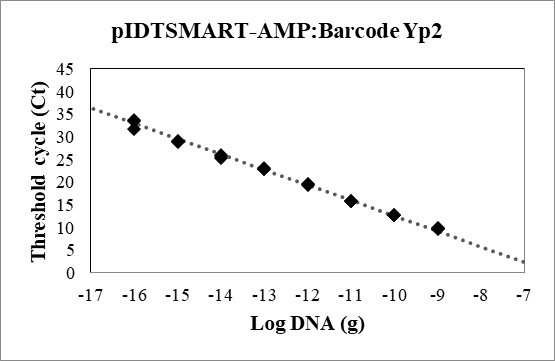

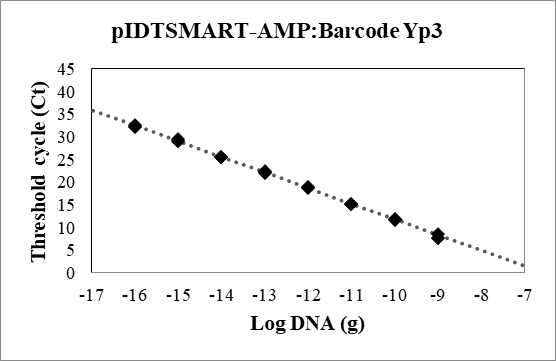

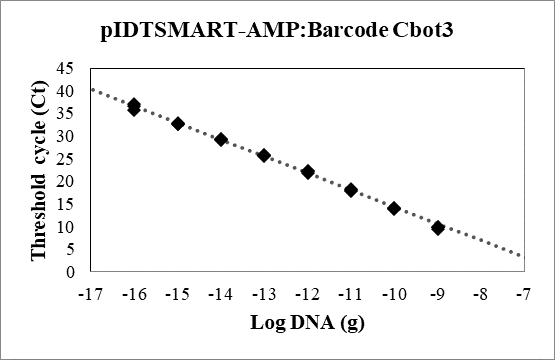


Figure S1. qPCR standard curves generated for the remainder of the 21 barcodes created by the barCoder algorithm. Template DNA is the indicated barcode in the pIDTSMART-AMP plasmid backbone. For each standard curve, data from three replicates and a trendline are shown.

⁂ Ct value not determinable for 3/3 replicates.
